# Anaesthesia for Minimally Invasive Cardiac Surgery

**DOI:** 10.3390/jcdd10110462

**Published:** 2023-11-15

**Authors:** Daniel Aston, Daniel Zeloof, Florian Falter

**Affiliations:** Department of Anaesthesia and Critical Care, Royal Papworth NHS Foundation Trust, Cambridge Biomedical Campus, Papworth Road, Cambridge CB2 0AY, UK; dzeloof@gmail.com (D.Z.); florian.falter@gmail.com (F.F.)

**Keywords:** cardiac surgery, minimally invasive, minimal access, anaesthesiology

## Abstract

Minimally invasive cardiac surgery (MICS) has been used since the 1990s and encompasses a wide range of techniques that lack full sternotomy, including valve and coronary artery graft surgery as well as transcatheter procedures. Due to the potential benefits offered to patients by MICS, these procedures are becoming more common. Unique anaesthetic knowledge and skills are required to overcome the specific challenges presented by MICS, including mastery of transoesophageal echocardiography (TOE) and the provision of thoracic regional analgesia. This review evaluates the relevance of MICS to the anaesthetist and discusses pre-operative assessment, the relevant adjustments to intra-operative conduct that are necessary for these techniques, as well as post-operative care and what is known about outcomes.

## 1. Introduction

While cardiac surgery is still predominantly performed via median sternotomy, a growing number of procedures are being performed that fall under the umbrella of minimally invasive cardiac surgery (MICS). First performed in the mid-1990s [1], MICS does not have a universally agreed definition. The Society of Thoracic Surgeons (STS) defines minimally invasive surgery as ‘any procedure not performed with a full sternotomy and cardiopulmonary bypass (CPB)’ [2], although some procedures (particularly some types of minimally invasive valve surgery) are performed using CPB. The American Heart Association (AHA) gives the definition of minimally invasive cardiac surgery as any procedure performed via a “small chest wall incision that does not include the conventional full sternotomy” [3]. A variety of different techniques are employed [4] with the unifying hallmark of a lack of full sternotomy [5]. Cardiopulmonary bypass is utilised for some procedures and avoided for others. Examples range from Minimally Invasive Direct Coronary Artery Bypass (MIDCAB) and Minimally Invasive Mitral Valve Surgery (MIMVS) to procedures carried out in the cardiac catheter laboratory (‘cath lab’) such as Transcatheter Aortic Valve Replacement (TAVR) and Transcatheter Edge-to-Edge Repair (TEER) of the mitral valve.

MICS is an attractive option for many patients, in part offering advantages over traditional surgery such as reduced post-operative pain, shorter hospital stay, and earlier return to normal life. There is also a smaller risk of wound infection, bleeding, respiratory complications, and atrial fibrillation [5,6]. Younger patients are especially keen on superior cosmetic outcomes [7]. However, there are drawbacks of MICS including procedure-specific complications, the considerable surgical training, and skill required, and the need for careful patient selection as minimally invasive procedures are not appropriate for all.

## 2. Pre-Operative Assessment and Patient Selection

The pre-operative anaesthetic assessment of a patient presenting for MICS includes the standard history, examination and investigations obtained prior to any cardiac surgery. Some areas require enhanced attention or special emphasis when a minimally invasive technique is to be used. A history of previous thoracic surgery, chest wall or rib abnormalities or some inflammatory disease processes may lead to the development of adhesions and make the minimally invasive approach to the heart difficult or impossible. Table 1 shows a list of conditions where alternatives to MICS should be considered carefully. Severe aortic regurgitation is listed as a contraindication to MIMVS; some techniques used for minimally invasive aortic valve replacement can be used in the presence of aortic incompetence, such as the use of rapid-deployment valve prostheses and in TAVR. Effective cardioplegia may sometimes be possible in MICS using retrograde delivery when aortic regurgitation renders the antegrade route ineffectual.

Symptoms and signs of the surgical cardiac lesion should be elicited, along with those of any other cardiac problems that may be additional surgical targets; the presence of multiple cardiac pathologies that all require surgical intervention is likely to make minimally invasive surgery a less feasible option. Echocardiographic assessment should pay particular attention to excluding the presence of poor ventricular function, aortic valve incompetence, mitral annular calcification, patent foramen ovale (PFO), and persistent left superior vena cava—all of which may make MICS significantly more challenging.

Evaluation of the vascular system as a whole is essential, especially if peripheral cannulation is planned for CPB [4]. CT angiography is often included as part of the standard pre-operative assessment for MICS [10]; aneurysm, significant tortuosity or atheroma, dissection or the presence of stents, grafts or other previous surgical repairs must be carefully assessed.

Some MICS procedures are performed via a small thoracotomy and many of these require one-lung ventilation (OLV) to give adequate surgical access to the heart. The patient may be supine or in a semi-lateral position. All these factors predispose to hypoxia and a history of chronic respiratory disease may compound this. Therefore, the threshold for performing Pulmonary Function Tests (PFTs) should be low. In patients presenting for thoracic surgery, a finding of a Forced Expiratory Volume in 1 s (FEV-1) and transfer factor (TLCO), both of which are greater than 40% of what was predicted, suggest the patient is at low risk of post-operative pulmonary complications, although patients with values below this should not necessarily be denied surgery as there may still be a significant chance of an acceptable outcome [11]. One small retrospective trial of patients with chronic obstructive pulmonary disease (COPD) undergoing cardiac valve surgery suggested more favourable outcomes in the group undergoing surgery using a minimally invasive technique, despite the use of OLV in this group [12].

The presence of pulmonary hypertension and right ventricular dysfunction needs to be assessed, as it is possible that the use of OLV may occasionally lead to complications including further increased pulmonary arterial pressure, raised right ventricular afterload, and cardiac failure in these patients [13].

The use of transoesophageal echocardiography (TOE) is widely regarded as essential for some MICS procedures (especially MIMVS) to the extent that a contraindication to TOE is often considered a contraindication to a minimal access approach [6,14]. Any suggestion of upper gastrointestinal pathologies such as known oesophageal webs, pouches or varices, active peptic ulcer disease, hiatus hernia, or previous surgery or radiotherapy to the neck and chest should be carefully reviewed and risks evaluated appropriately.

The planned position of the patient on the operating table should be considered, and it is likely to be challenging to place patients with chest wall deformities, kyphoscoliosis, or other significant musculoskeletal pathology into a posture that offers sufficient surgical access. Orthopaedic problems with the ipsilateral upper limb that preclude a position allowing access to the hemithorax may make the surgical approach difficult. Some positions may predispose to brachial plexus injury due to tension on the arm, and measures should be taken to minimise this risk.

Patients with significant obesity present particular challenges for the surgeon and anaesthetist. In addition to the difficulty in positioning these patients, it may be difficult to properly site a double-lumen endotracheal tube, they may tolerate OLV less well and they may require an additional venous drainage cannula to allow adequate flow during CPB.

## 3. Relevant Surgical Principles and Techniques

The surgical principles clearly depend largely on the procedure to be performed, as does the surgical approach. Aortic valve surgery is usually approached via a ministernotomy or right anterior thoracotomy, while the mitral valve is accessed using the right mid-axillary approach.

TOE-guided positioning of cannulae inserted via the femoral artery and veins is the most common strategy to facilitate CPB. In contrast to conventional cardiac surgery via full sternotomy, the venous cannula is inserted first in MICS [6]. If right atriotomy is required, two drainage cannulae will be required; the inferior vena cava (IVC) cannula is inserted via the femoral vein as discussed above, but the superior vena cava (SVC) cannula may need to be sited percutaneously via the right internal jugular (RIJ) vein. This can be alongside the central venous catheters, or these can be sited in the left internal jugular vein instead.

Arterial return flow is usually achieved using a cannula inserted using the Seldinger technique via the femoral artery. However, end-to-side grafting of a dacron graft to the femoral artery, or direct cut down onto the artery are alternative techniques.

Cross-clamping the aorta can be achieved in a similar way to open surgery, under direct vision using a transthoracic Chitwood aortic clamp or via an endoaortic balloon-tipped catheter (endoballoon) [15]. The former is the technique more familiar to most surgeons, allows the use of reusable clamps and is inexpensive. However, the presence of the clamp clutters the already small surgical field further and the technique necessitates a puncture in the aortic wall for infusion of cardioplegia. This must then be closed at the end of surgery and carries a risk of bleeding.

The endoballoon occludes the ascending aorta and simultaneously allows an infusion of cardioplegia, root venting, and pressure monitoring through a central lumen in the catheter, which opens proximal to the balloon. This reduces the number of instruments in the surgical field and eliminates the need for a cardioplegia cannulation site in the aorta. Disadvantages of the endoballoon include its single-use (and therefore expensive) nature, and the fact that it can migrate both proximally and distally during the procedure to obscure the surgical field or occlude arterial flow via the innominate artery to the upper body and head, respectively. Additionally, the endoballoon is usually passed through the arterial cannula which can reduce flow and lead to high line pressures. If the aortic line pressure is higher than 300 mmHg, the endoballoon is usually inserted via separate cannulation of the contralateral femoral artery [15].

There have been no prospective randomised controlled trials comparing the use of endoballoon with traditional cross-clamping, but retrospective data does not demonstrate an advantage of one technique over the other in terms of the degree of cardioprotection or incidence of bleeding [16,17]. There is some evidence that the use of the endoballon carries a higher risk of aortic dissection [18,19]. The use of retrograde cardioplegia is unusual in MICS due to the technical difficulty in siting a coronary sinus (CS) catheter through the small surgical field. That said, a catheter can be placed either via the right internal jugular vein when using the HeartPort, or via the right atrium in other cases. The technique is the same in both cases and relies on guidance from the TOE the pressure waveform transduced from the catheter tip. The limiting factor in both cases is the lack of a stabilising hand behind the heart and it can on occasion be impossible to place the CS catheter in an appropriate position.

Management of CPB follows the same principles as those used in conventional cardiac surgery.

## 4. Intra-Operative Anaesthetic Management

Differences from standard cardiac surgical setup include the use of a double-lumen endotracheal tube, and the mandatory placement of external defibrillator pads as internal cardioversion is often not possible during MICS.

### 4.1. Monitoring

Monitoring standards are not dissimilar to those for standard cardiac surgery.

The planned use of an endoballoon mandates the use of bilateral radial arterial pressure monitoring, as the loss of perfusion to the right side suggests distal migration of the balloon and allows prompt correction. The pressure at the distal tip of the endoballoon is also monitored.

The use of cerebral oximetry may also allow the detection of poor perfusion of one or both sides of the upper body. This may occur if the endoballoon migrates but can also be seen if the heart is allowed to eject blood while there is no ventilation and the CPB return flow is distal to the innominate artery. The perfusion of the upper body with deoxygenated blood while the lower body enjoys oxygenated perfusion is known as Harlequin syndrome and may lead to life-threatening cerebral hypoxia [20].

The use of OLV and lung isolation may lead to high impedance of cardiac electrical signals to the skin surface and a poor electrocardiograph trace. Modification of the standard ECG electrode positions may be required to avoid this problem.

### 4.2. Induction and Maintenance

Anaesthetic induction proceeds as for any cardiac surgery. The patient is intubated with a double-lumen endotracheal tube (DLT), and the position is checked—usually using a bronchoscope. Alternatively, a single-lumen tube is used with a bronchial blocker used to isolate the lung. Prophylactic antibiotics are given. Maintenance of anaesthesia is commonly achieved using an intravenous agent.

### 4.3. Positioning

The majority of procedures are performed through a mini-thoracotomy, often requiring OLV to facilitate access to the heart, or through a partial sternotomy [21]. For the former, the patient is positioned with one hemithorax raised by a gel pad or inflatable bag to open the anterior intercostal spaces while the arm is secured away from the surgical site and pressure points must be carefully padded to avoid tension on the brachial plexus. The latter is performed in the supine position.

As for any anaesthetic, routine care must be taken to ensure that the cervical spine and upper and lower limb joints are in a neutral position and that the eyes and other pressure points are adequately protected.

### 4.4. TOE

The TOE is used to perform a comprehensive study at the beginning of the case. In particular, the diagnosis and surgical target should be confirmed, and aortic regurgitation excluded or quantified. The diameter of the ascending aorta and aortic root should also be measured if using the endoballoon. The integrity of the interatrial septum should be assessed.

During cannulation for CPB, TOE is used to help guide the position of the cannulae. Venous cannulation is best seen in the bicaval view, and guidance of the cannula position helps to reduce the risk of complications such as malposition in the hepatic vein or interatrial septum perforation. Unless right atriotomy is required during surgery, the tip of the venous drainage cannula should be in the proximal SVC. If there is a need to open the atrium, then the IVC cannula should be positioned at the cavoatrial junction and a separate SVC cannula must be sited.

If an endoballoon is to be used, TOE can be used to guide its position. The tip should be placed in the ascending aorta. As the balloon is inflated the pressure causes an indentation on the aortic wall. The flow of cardioplegia into the aortic root can be seen using TOE and can be followed into the coronary ostia. Lack of regurgitation of cardioplegia into the left ventricle (LV) can also be confirmed. It is important to visualise the LV while cardioplegia is given. Application of the endoballoon can lead to distortion of aortic valve anatomy and subsequent ventricular distention should the valve become incompetent. Venting of the aortic root is also possible using the endoballoon.

Finally, since it is not possible for the anaesthetist or surgeon to easily see the right ventricle—as it is during conventional cardiac surgery—TOE provides information regarding volume status and right ventricular (RV) function. This is particularly useful during the process of separation from CPB.

### 4.5. Separation from CPB

Separation from CPB is not significantly different to the process that occurs during conventional cardiac surgery. There are several reasons why it may be beneficial to temporarily ventilate using both lungs during weaning from CPB:

1. Two-lung ventilation will allow superior gas exchange; normoxia and the absence of respiratory acidosis will promote better cardiac function;
2. The isolated and collapsed lung presents a higher pulmonary vascular resistance (PVR) to the RV than a normally ventilated lung, while ventilating both lungs is likely to improve right ventricular function by optimising PVR;
3. Ventilation of both lungs will enhance the mobilisation of air collections in the pulmonary veins and aid with deairing the heart. As is the case with conventional cardiac surgery, TOE can be used to guide deairing.

Following successful separation, it is often necessary to isolate the lung again so that surgical haemostasis can be ensured.

### 4.6. Analgesia

Like conventional cardiac surgery, intraoperative analgesia for MICS relies heavily on opioids, although opioid-sparing and opioid-free approaches have been described [22]. In light of modern approaches such as Enhanced Recovery after Surgery (ERAS), the side effects of opioids and their effect on recovery [23] should be considered and the doses used moderated [24]. Other available modalities of analgesia should also be considered. These include non-opioid pharmacological agents such as ketamine and clonidine and specific local anaesthetic nerve blockade. The latter is particularly useful in MICS and may aid in achieving intra-operative haemodynamic stability. In selected cases, good regional analgesia may allow extubation of patients in the operating room at the end of surgery and therefore lead to a significantly reduced length of stay in intensive care.

Thoracic wall regional nerve blockade can be provided both pre or post-operatively with paravertebral, serratus anterior plane, erector spinae and Pectoralis (PEC) I + II blocks. In some instances, a local anaesthetic can be placed under direct vision by the surgeon (e.g., intercostal nerve blockade) [10]. Analgesia using local anaesthetic can also be provided with continuous infusions, and catheters may be placed using ultrasound guidance (e.g., for paravertebral block [25]) or under direct vision by the surgeon (e.g., intrapleural block [26]). It is important to consider that in patients with ongoing infusions of local anaesthetic, pain that would otherwise indicate the presence of a significant surgical complication may be masked and therefore a high index of suspicion is required.

## 5. Post-Operative Management

In selected cases, it may be appropriate to extubate patients who have had MICS before leaving the operating theatre. In most cases, however, admission to a cardiothoracic intensive care unit will be required. If a DLT has been used for OLV during surgery, this should be exchanged for a standard single-lumen tube.

Post-operative monitoring post MICS follows the same principles as following conventional cardiac surgery, with some important caveats. The first is that bleeding may be more concealed in MICS than following full sternotomy, and the development of haemodynamic instability should prompt early investigation to rule out tamponade or the development of other covert haemorrhages. If cannulation for CPB has been performed peripherally, there should be vigilance against haematoma formation and development of peripheral limb ischaemia.

## 6. Redo Surgery

Minimally invasive approaches to cardiac valve surgery are increasingly being used where reoperation is required as an alternative to resternotomy. Resternotomy carries an increased risk of damage to structures such as patent coronary grafts due to distorted anatomy caused by adhesions and scarring [27]. It is also associated with prolonged CPB and cross-clamp times as well as higher transfusion requirements compared to first-time cardiac surgery. The theoretical appeal of minimally invasive redo surgery is therefore the minimisation of trauma and avoidance of the challenging dissection planes. This needs to be carefully balanced with the concern over suboptimal myocardial protection due to the smaller surgical field and the difficulty of successfully isolating any internal mammary artery grafts during aortic cross-clamping and myocardial ischaemia [28].

Despite these concerns, one meta-analysis comparing minimally invasive redo aortic valve replacement (MIrAVR) with conventional redo surgery has shown no significant difference in mortality, risk of stroke, rates of permanent pacemaker implantation, renal failure, re-operation for bleeding, or length of hospital stay [29]. Perhaps unexpectedly, cross-clamp and CPB times were similar in this study too. Reassuringly, in this review, myocardial protection was achieved without occlusion of the IMA and by utilising deeper hypothermia and there were subsequently no reported post-operative myocardial infarctions.

Two recent meta-analyses comparing MIMVS vs. conventional resternotomy for redo mitral valve surgery showed an improved mortality rate in patients who had a minimally invasive approach rather than resternotomy [30,31]. A recent analysis of patients having mitral surgery after a previous sternotomy in the national Netherlands heart registry showed no difference in thirty-day mortality or five-year survival, although there was a lower incidence of prolonged intubation and new-onset arrhythmia in the minimally invasive group [32]. There have so far been no large randomised controlled trials and therefore these meta-analyses were only able to use retrospective non-randomised data.

## 7. TAVR

MICS includes many percutaneous cardiac interventions that are less invasive than surgery and can be performed in the cath lab without CPB. The most commonly performed of these is transcatheter aortic valve replacement (TAVR).

TAVR via the transfemoral approach is the most frequently used technique. However, in some patients, other approaches may be utilised, including trans-axillary (TAX) and, less commonly, trans-carotid (TC), trans-apical (TA), and trans-caval (TCV). Factors that are associated with increased vascular complications, and therefore favour a non-femoral approach, include small iliofemoral artery calibre or the presence of vessel aneurysm or severe atheroma, significant arterial wall calcification and degree of tortuosity [33]. TAX is usually the second most preferred approach.

The approach to TAVR is one of the principal factors used to decide on the anaesthetic technique. In a high proportion of cases, transfemoral TAVR may be achieved with local anaesthesia and conscious sedation while other approaches will often require a general anaesthetic.

One important consideration is the proximity of the surgical field to the airway. For this reason alone, TAX and TC approaches are likely to warrant general anaesthesia. The ability of the patient to tolerate the procedure under sedation must also be taken into consideration. Emergent conversion to general anaesthetic can be challenging even for the experienced anaesthetist due to the ergonomics of the cath lab and the physiology of the patient with severe aortic valve disease. If TOE is likely to offer significant advantages over transthoracic echo (which is the mainstay of ultrasound imaging used during TAVR) for a particular patient, general anaesthesia facilitates this.

Routine monitoring for TAVR is not dissimilar to that used for standard cardiac surgery if the procedure is being performed under general anaesthesia. Invasive arterial pressure monitoring is usually established using a left-sided arterial line although the laterality of lines should be discussed, as the cardiologist may need to insert an angiography catheter into a radial artery. In cases where TAVR is being performed under conscious sedation, invasive monitoring may be established by the cardiologist. Central venous pressure monitoring is not commonly used, although central venous access should be available regardless of the anaesthetic technique so that vasoactive medications may be administered if required.

Recognised complications that the anaesthetist should be aware of include those due to poor positioning of the valve that may lead to haemodynamic compromise, such as para-valvular regurgitation (3–4%) [34] or due to occlusion of a coronary ostium (<1%) [35] and myocardial ischaemia [36]. Self-expanding TAVR valves (e.g., Edwards Core-valve) can be captured and redeployed in the event of suboptimal positioning. Patients with a pre-existing poor LV function are particularly susceptible to haemodynamic compromise that may persist beyond valve deployment. Major vascular complications occur in 2–3% [34]. Very rarely, the valve may embolise leading to distal ischaemic consequences. Approximately 9% of patients will require a permanent pacemaker [34], particularly if there is a pre-existing right bundle branch block [37]. Neurological complications such as stroke are a recognised risk of TAVR, occurring in approximately 2% of patients [38]. The BHF PROTECT-TAVI study is a large ongoing randomised controlled trial studying whether the use of cerebral embolic protection (CEP) devices during the procedure will reduce this risk [39].

Transcatheter procedures are significantly less painful than open procedures and often require little more than local infiltration, ultrashort-acting opioids, and paracetamol. An exception to this is the TA TAVR as this relies on a mini-thoracotomy [40]. Paravertebral blocks have been shown to provide effective analgesia for this procedure and at the authors’ institution PECS and serratus anterior blockade are routinely utilised to augment general anaesthesia [41].

Post-operative pain can be managed with supplemental oral or IV analgesia. It is prudent to consider the probability of the pain being the first presentation of a hitherto unrecognised occult complication (e.g., tamponade, bleeding, etc.) before prescribing patient-controlled analgesia.

## 8. Outcomes

The outcomes of MICS compared with conventional cardiac surgery performed via a full median sternotomy have been studied, although in some cases more randomised data are needed. In general, the overall surgical, bypass and cross-clamp times tend to be longer with MICS [42,43,44]. However, rates of mortality, kidney injury, post-op atrial fibrillation, and stroke are generally not significantly different [42,45,46,47,48], while pain scores, patient satisfaction, and ICU length of stay have been reported as improved when compared with conventional surgery [42,43,45,49].

### 8.1. Mitral Valve

There has been a lack of high-quality randomised trials comparing outcomes of MIMVS with conventional sternotomy for mitral valve surgery (CMVS), especially in the longer term. A recent systematic review and meta-analysis comparing 119 studies showed a reduced requirement for blood transfusion and a shorter length of hospital stay associated with MIMVS compared with CMVS. Some non-randomised retrospective studies suggested a lower mortality associated with MIMVS, but this finding was not supported by randomised prospective trials [50]. Earlier meta-analyses showed similar findings and suggested reduced pain and faster recovery following MIMVS, despite longer cardiopulmonary bypass and cross-clamp times [44,45,51,52].

A recent analysis of the Mini-mitral International Registry separated patients having MIMVS by surgical risk according to their EuroSCORE-II (ESII). Operative results were considered excellent in all categories except those whose ESII was 12% or over. Overall, the observed mortality was found to be lower than expected in all risk groups [53].

### 8.2. Aortic Valve

A plethora of studies have compared traditional surgical aortic valve replacement (SAVR) with TAVR. A 2019 meta-analysis [54] included seven trials with over 8000 patients comparing TAVR with SAVR for severe aortic stenosis. When transfemoral TAVR was used, a relative reduction in all-cause mortality of 17% was seen up to two years post procedure. Additionally, those undergoing TAVR had a lower risk of stroke, acute kidney injury, major bleeding and new-onset atrial fibrillation when compared with those having a SAVR. These advantages were seen regardless of the type of TAVR used and of the surgical risk cohort.

However, indications for SAVR remain [55]. Patients with bicuspid aortic valves, unfavourable aortic root anatomy, significant subannular calcification or peripheral vascular disease are still thought to be more suited to SAVR than TAVR, as are patients with or post-endocarditis and those who require multiple valve procedures. Questions also remain regarding how appropriate TAVR is for younger patients in whom the durability of the valve replacement is of increased importance.

High-quality studies comparing conventional SAVR with other minimally invasive techniques such as mini-sternotomy or anterior right thoracotomy for aortic valve procedures have generally been lacking and there has been slow surgical uptake of these techniques, being used in only 12% of SAVRs in the UK and the US in the 12 years to 2018 [56]. However, there is some evidence to suggest they carry some advantages. In one meta-analysis including data from 50 studies and more than 12,000 patients, minimally invasive AVR (MIAVR) was associated with reduced requirement for blood transfusion, shorter length of intensive care stay, less pain and lower incidence of renal failure compared with conventional SAVR. On closer analysis, the study demonstrated that these advantages tend to be a feature of the mini-sternotomy but not the mini-thoracotomy approach to AVR. There was no difference in rates of mortality, stroke, respiratory failure, and re-look surgery for bleeding or deep wound infection when compared with conventional SAVR [57]. Similarly, a small study comparing MIAVR with full sternotomy in patients over 80 years of age who required redo aortic valve surgery demonstrated improved survival at 5 and 8 years [27]. A larger subsequent meta-analysis of patients having redo aortic valve surgery could not confirm a difference in in-hospital mortality, but did not have sufficient data to compare outcomes for a longer period of time after surgery [29].

## 9. Future Directions and Emerging Applications of MICS

Mechanical support is another area of cardiac surgery where a minimally invasive approach is showing increasing promise. A small non-randomised prospective study [58] of patients over 60 years of age with congestive cardiac failure had a left ventricular assist device (LVAD) implanted as destination therapy either using a conventional open surgical technique or using a minimally invasive technique. There was no difference in 2-year survival, but the latter group had a lower incidence of post-op bleeding and lower rates of post-operative extended inotropic support. One hypothesis proposed as an explanation for this latter finding was that the minimally invasive approach allowed LVAD implantation while preserving the majority of the pericardium and therefore maintaining the natural limits of the right ventricle. This avoided over-distension and better safeguarded right ventricular function [58].

## 10. Conclusions

In summary, MICS is a diverse range of techniques that in many cases are closely aligned with the principles of enhanced recovery. Post-operative pain and recovery times are reduced, which translates into reduced length of intensive care and hospital stay. Patient satisfaction is therefore increased. Success depends upon careful patient selection and an institutional learning curve is to be expected. However, many aspects of these techniques remain to be proven and high-quality randomised trials are still needed to better delineate which approaches offer the best outcomes for patients.

It is essential that anaesthetists are familiar with minimally invasive cardiac surgical techniques, along with the challenges they present and the changes to practice that they demand. Anaesthetists must have the skills to carry out a thorough pre-operative assessment, maintain a high degree of attentiveness and communicate clearly with the surgical team. They must be able to perform high-quality TOE and be prepared to provide multi-modal analgesia. These skills will help to ensure that the best surgical results are obtained while risks are minimised.

## Figures and Tables

**Table 1:** Contraindications to minimally invasive valve surgery (adapted from [8,9]).

Aortic Surgery	Mitral Surgery
Severe calcification of the aortic root and/or ascending aorta	Severe aortic regurgitation
	Severe mitral annular calcification
Significant tortuosity of iliac or femoral arteries if femoral access for CPB planned	
Morbid obesity	
Severe chest wall deformities or previous radiotherapy to the chest	
Mobile aortic atheroma	
Absolute contraindication to TOE	

## Data Availability

Data sharing not applicable.
